# Can a Novel Light Weight Minimal Support Lifting Exoskeleton Modify Lifting Movement in People without Low Back Pain?

**DOI:** 10.3390/s24155067

**Published:** 2024-08-05

**Authors:** Tamer Burjawi, Rifai Chai, Matthew Arrowsmith, Adrian Pranata

**Affiliations:** 1 Department of Health Sciences and Biostatistics, Swinburne University of Technology, Melbourne, VIC 3122, Australia; 2 Department of Engineering Technologies, Swinburne University of Technology, Melbourne, VIC 3122, Australia; rchai@swin.edu.au (R.C.);; 3 School of Health and Biomedical Science, RMIT University, Bundoora, VIC 3082, Australia; adrian.pranata@rmit.edu.au

**Keywords:** low back pain, lifting, exoskeleton, movement, kinematics

## Abstract

Low back pain (LBP) is a major contributor to lifting-related disabilities. To minimize the risk of back pain, emerging technologies known as lifting exoskeletons were designed to optimize lifting movements. However, it is currently unknown whether a minimally supportive exoskeleton can alter the lifting movement in people without LBP. This study aims to investigate if wearing a novel lightweight exoskeleton that minimally supports the back, hip, and knee can alter the lifting range of motion and movement variations in people without LBP. This study also aims to investigate if wearing this novel exoskeleton can result in a reliable between-day lifting movement. In two separate sessions (each one week apart), fourteen participants lifted a box (that weighed 10% of their body weight) ten times, once while wearing an exoskeleton and once while not wearing an exoskeleton. Wearing the novel exoskeleton during lifting produced moderate-high, test-retest reliability (Trunk: ICC_3,1_ = 0.89, 95% CI [0.67, 0.96], SEM = 9.34°; Hip: ICC_3,1_ = 0.63, 95% CI [0.22, 0.88], SEM = 2.57°; Knee: ICC_3,1_ = 0.61, 95% CI [0.23, 0.87], SEM = 2.50°). Wearing an exoskeleton significantly decreased the range of motion of the knee (F_1,4_ = 4.83, *p* = 0.031, ηp^2^ = 0.06). Additionally, wearing an exoskeleton significantly decreased hip (diff = 8.38, *p* = 0.045) and knee (diff = −8.57, *p* = 0.038) movement variability; however, wearing an exoskeleton did not decrease the movement variability of the body’s trunk (diff = 0.60, *p* = 1.00). Therefore, minimally supported lifting through the use of exoskeletons can modify movement in people without LBP and produce reliable lifting movements. Wearing the novel exoskeleton is also desirable for monitoring lifting movements. Future studies should investigate the use of sensors and IMU to monitor lifting movement at work with the least amount of intrusion on an individual’s movement.

## 1. Introduction

Low back pain (LBP) is the leading cause of disability globally [1], affecting 77% of Australians who are of working age (15–64 years old) annually. In particular, LBP is common (21.9% chance of recurrence) in manual laborers [2]—especially in those whose work involves repeated manual lifting [3]. Continuous lifting for a prolonged period of time day in and day out is detrimental to musculoskeletal health [3]. The cyclic load encourages repeated movement to increase stress on the back triggering back-related disorders [4]. Therefore, assessing limiting movements, including range of motion (ROM), and evaluating movement variability may prove desirable to reduce the risk of back injuries. In order to decrease the risk of developing occupational LBP, lifting movement assessments and modification are a focus of occupational rehabilitation [5,6]. However, modifying lifting movements (e.g., the recommendation to lift with the legs and keep the back straight) has not been associated with meaningful changes in LBP recurrence at work [7]. In part, this could be due to the complex and multifaceted nature of LBP, which shows variability in the presentation of its pain symptoms. Symptoms of LBP are distinguished by the spatial location of pain and the duration of symptoms. The location of LBP is presented in body regions affected by the sciatic nerve. This includes the lumbar region of the spine (i.e., from costal margins of the twelfth rib along the iliac crest) and the lower limbs (i.e., from the gluteus muscle and hamstrings to the lowest portion of the calves and sole of the foot) [8]. The duration of LBP must remain for a period of six weeks or less to be defined as acute, six to twelve weeks to be defined as subacute, and twelve weeks or longer to be defined as chronic [9]. Despite improvements following acute and subacute LBP, symptoms of LBP can reoccur one year after the initial presentation and continue to persist for longer than three months [10]. To propose a new intervention that aims to minimize the symptoms of LBP, it is crucial to understand the factors that encourage the progression to LBP. An impaired trunk and lower limb movements are commonly observed in people with LBP compared to healthy people. It has been demonstrated in the literature that people with LBP can have increased [11] or decreased [12] trunk movement during bending and lifting when compared to healthy people without LBP. Additionally, there are studies that suggest people with LBP utilize less [13] or more [14] of their hip’s range of movement during bending forward than healthy control ranges that have been defined in the literature. Altered trunk and hip movement coordination is typically estimated and targeted for rehabilitation for people with LBP via corrective postural exercise prescription in a clinical setting [15]; however, the size of the effect from these exercises is small [16]. In part, the limited effects could be due to the inherent limitations of the visual observation strategy that is employed by clinicians to accurately quantify lumbar spine and hip movements during bending (and lifting) assessments. Therefore, a kinematic analysis of the trunk and legs is necessary for the accurate assessment of altered movement in people with LBP. This commonly involves the assessment of lifting biomechanics.

Lifting is a daily occupational task involving complex trunk, hip, and knee coordination [17]. The performance of safe lifting is dependent upon several factors such as speed of movement, position of the feet relative to the object lifted, physical factors such as hamstring tightness, and lifting technique [18,19]. A suboptimal lifting technique and high lifting frequency have been associated with lower back injuries [20]. Ref. [21] found that repetitive trunk flexion (i.e., twenty-five times or more per day) and rotation of the body when lifting a heavy object (i.e., 25 kg) in an occupational setting are both associated with excessive loading of the lumbar spine [22], which may result in the development of LBP. Spinal loading is when shear force along the spine compresses the intervertebral discs at the lumbar level due to trunk movement [23]. Previous studies indicate that lumbar loading was more closely associated with trunk flexion movement than with lifting a heavy weight [24].

To decrease the risk of developing occupational LBP, lifting movement assessments and modifications are a focus of occupational physiotherapy sessions [5,6]. However, modification suggestions for lifting movements (e.g., the recommendation to lift with the legs and back straight) have not been associated with meaningful changes in LBP recurrence at work [7]. In part, this is due to the lack of technology to assess and modify lifting movement accurately in clinical settings. Therefore, novel technologies need to be developed to improve lifting safety at work.

In recent years, there has been a rise in the development of new technologies, such as lifting exoskeletons, to assist manual workers with lifting tasks at work [25]. A lifting exoskeleton is a wearable frame worn to support and guide lifting movements and potentially offload the lumbar spine and legs. Lifting exoskeletons have multiple types that are distinguished according to their characteristics. They are classified as powered (otherwise known as active) or passive exoskeletons. Passive lifting exoskeletons are comprised of materials (e.g., springs, elastic bands, and flexible components) that store and release energy from the user’s lifting movement. Current studies demonstrated equivocal changes in lifting techniques when wearing a passive and powered exoskeleton [26,27]. Existing lifting exoskeletons that have been researched in the literature provide support mainly to trunk join movement. However, there is a paucity of evidence in the research associated with the exoskeleton that supports leg joint movements, especially the knee. Due to the muscle geometry and ability to generate extension force, the hip extensor muscles (e.g., gluteus maximus, hamstring) are the primary muscles of lifting [28]. The lumbar extensor muscles (e.g., lumbar multifidus) provide inter-segmental compression to minimize or control movement of the lumbar spine [29]. Therefore, it is apt to provide support to guide hip and knee movements in lifting. This can be used to monitor movements via movement sensors or Inertial Measurement Units (IMUs). As this type of exoskeleton is light, there is no added extra weight, and may allow people to lift comfortably for prolonged use daily (i.e., to monitor movement and loading). However, it is still unclear how much support is required by an exoskeleton to modify lifting and whether wearing an exoskeleton providing minimal support to back and leg joints can yield a reliable lifting movement. Thus, it is imperative to discover if a lightweight minimal support exoskeleton can lead to significant changes in movement.

Therefore, this study aims to investigate the effects of wearing a novel passive exoskeleton on lifting movements in people without LBP. In particular, this study aims to: (1) investigate whether the use of a minimally supportive exoskeleton yields reliable between-day lifting techniques in people without LBP (seven days apart); (2) investigate whether wearing a minimal support exoskeleton can modify the ROM of the trunk, hip, and knee (i.e., lifting technique) during a lifting task when compared to lifting without an exoskeleton in people without LBP; and (3) investigate whether wearing a minimal support exoskeleton can modify trunk, hip and knee movement variations (i.e., an additional component of lifting technique) during a lifting task when compared to lifting without an exoskeleton in people without LBP. We hypothesized that (H_1_) the use of a minimal support exoskeleton will yield reliable between-day ROM of the trunk, hip, and knee during lifting techniques in people without LBP (H_2_) wearing a minimal support exoskeleton will result in no significant changes in the ROM of the trunk, hip, and knee when compared to lifting without an exoskeleton in people without LBP and (H_3_) wearing a minimal support exoskeleton will not result in significant changes in the trunk, hip and knee movement variability when compared to lifting without an exoskeleton in people without LBP.

## 2. Materials and Methods

The current study conducted an observational study to assess the test-retest reliability of lifting movements and a case-control study to investigate the changes in lifting movements while the participants lifted without and with an exoskeleton.

### 2.1. Participants

A total of fourteen participants (n*_female_* = 3) aged 18–65 years old without a history of LBP were recruited from Swinburne University of Technology, Hawthorn, Australia. Participants were included in this study if they reported no previous history of trunk and lower limb injury or surgery, were in good health, and were not pregnant. Participants were excluded from this study if they reported previous history of trunk or lower limb injuries or surgery, serious health issues (e.g., cancer, active rheumatoid arthritis, significant/unmedicated heart condition), were pregnant, and had poor English language competency (verbal and written). Eligible participants were invited to attend two laboratories one week apart. This study has received ethics approval from Swinburne University’s Human Research Ethics Committee (ID: 20226292-10122). Informed consent was obtained from all participants included in the study. All procedures performed in studies involving human participants were in accordance with the ethical standards of the institutional and/or national research committee.

### 2.2. Exoskeleton Tested

**Figure 1:**
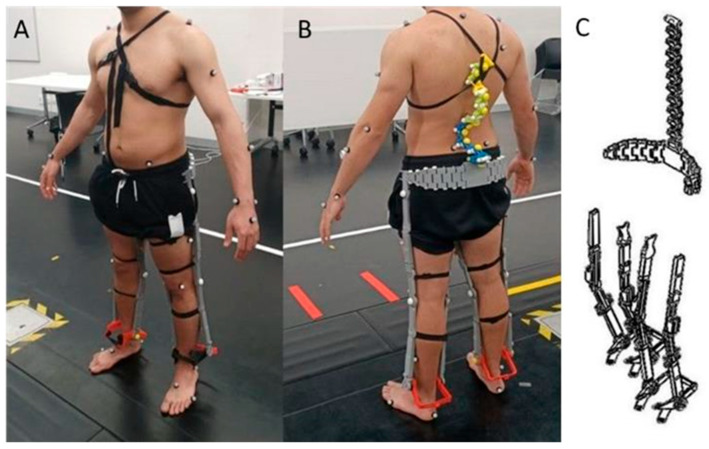
The novel lifting exoskeleton was utilized in this study. The anterior view of the exoskeleton (**A**), the posterior view of the exoskeleton (**B**), and the lifting exoskeleton schematics (**C**).

The novel lifting exoskeleton utilized in this study was 3D-printed (Zortax M200, Poland). It was made of Acrylonitrile Butadiene Styrene plastic and weighed 2.5–3.0 kg. It comprised eight lower limb components on each leg (i.e., for the hip one component attached to the medial thigh and one component to the lateral thigh, for the knee one component attached to the medial shank and one component attached to the lateral shank which can be merged and secured together with a thumb screw) and one spinal component that extended from the pelvis to the inferior angle of the scapula. It has ten individual components. Components are attached using elastic and Velcro straps forming an X across the chest cantered around the sternum which allows freedom of movement in the trunk, hip, and knee. Furthermore, other attachments include one strap on each foot, one strap on each shin, two straps on each thigh (extending from above the knee to below the groin), and one strap across the waist. The novel minimal support exoskeleton used in this study is illustrated in Figure 1.

### 2.3. Procedure

**Figure 2:**
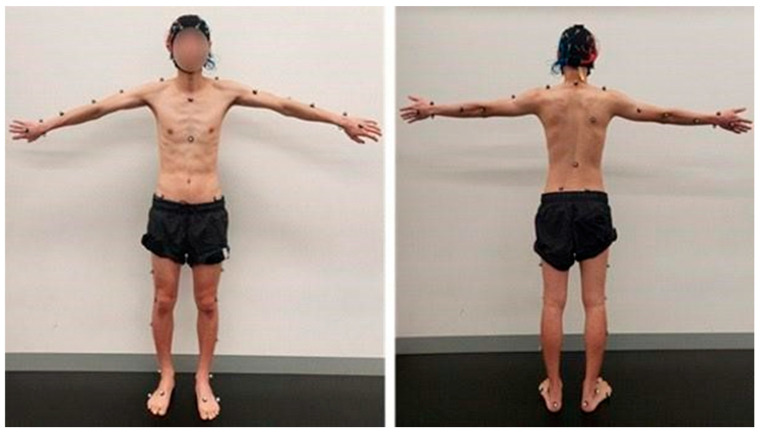
Marker orientation for laboratory lifting kinematic data capture.

Participants were instructed to lift a box (L × H × W = 36 × 32.5 × 36 cm) containing a load of 10% of the participant’s body weight, from the floor to an upright position with the box at the abdominal level using a technique of their own choosing. The lifting task was performed in view of a twelve-camera, infrared motion capture system with a capture frequency of 100 Hz (Qualisys, Stockholm, Sweden). Forty-one individual reflective markers were placed in areas of the head (four markers), upper limb (fourteen markers), trunk (nine markers), and lower limbs (14 markers) for kinematic data capture based on a previously published study [30]. The marker placements for this study are demonstrated in (see Figure 2).

The lifting task was performed over two conditions: (1) lifting without an exoskeleton and (2) lifting while wearing an exoskeleton. The participants were instructed to repeat the lifting task for ten repetitions for each lifting condition. Participants were provided a five-minute break in between each condition. A total of twenty lifts were performed by each participant.

### 2.4. Data Analysis

Range of motion (ROM) of the trunk, hip, and knee, analyzed in degrees (o), was calculated by deducting the minimum joint displacement angle from the maximum joint displacement angle for the trunk (angle formed between the thorax and pelvis), hip (angle between pelvis and femur) and knee (angle between femur and tibia) respectively. Flexion and extension movements were denoted in negative and positive values respectively. Lifting time was analyzed in seconds (s). Lifting speed was analyzed in degrees per second (o/s). Movement variability of the trunk was analyzed by calculating the standard deviation of the joint displacement of the trunk, hip, and knees in degrees [17,31]. Lifting time was calculated as lifting task completion time minus lifting task commencement time. All kinematic data were collected and cleaned using the Qualysis software version 2021.2(6940) (Qualisys AB, Göteborg, Sweden). These data were then processed using Visual3D v6.01.34 (CMotion, Germantown, MD, USA) using a custom-made pipeline before statistical analyses.

### 2.5. Statistical Analysis

Comparisons between the ROM of the trunk, hip, and knee with and without an exoskeleton will be assessed using the 3 (body regions) × 2 (time points) Repeated Measure Analysis of Variance (ANOVA). The ANOVA effect size was reported in partial eta squared (ηp^2^). Tukey Honestly Significant Difference test was utilized to analyze significant ANOVA interactions. The test-retest reliability of ROM of the trunk, hip, and knee will be analyzed using the Intraclass-correlation coefficient (ICC_3,1_), Bland–Altman plots, Standard Error of Measurement (SEM), and 95% Confidence Interval (CI). The ICC values of 0.0 to 0.5 were classified as weak, 0.5 to 0.75 as moderate, and 0.75 to 1.00 as a strong correlation [32,33]. The significance level was set at 0.05 for all analyses. All analyses were conducted using SPSS Statistics version 26 (IBM Corp., Armonk, NY, USA).

## 3. Results

All fourteen participants completed the study. The participants’ characteristics and descriptive statistics of the participant kinematic data can be found in Table 1 and Table 2, respectively.

**Figure 3:**
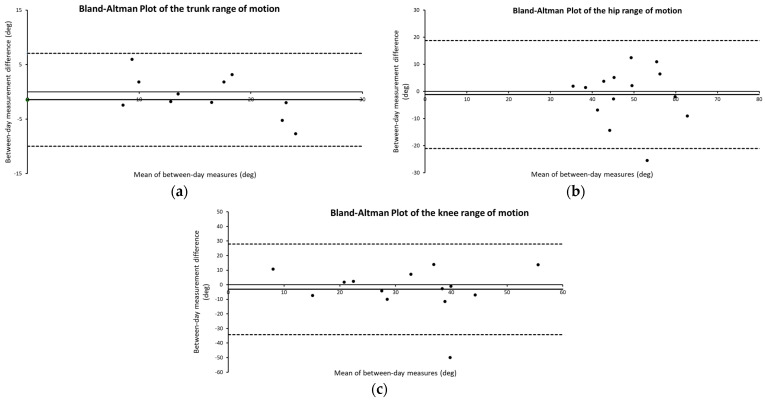
The Bland–Altman plots for the between-day assessment of the trunk (**a**), hip (**b**), and knee ROM (**c**).

The Bland-Altman plots for the trunk, hip, and knee test-retest sessions are shown in Figure 3. The test-retest reliability of the trunk ROM was strong (ICC_3,1_ = 0.89, 95% CI [0.67, 0.96], SEM = 9.34°), hip ROM was moderate (ICC_3,1_ = 0.63, 95% CI [0.22, 0.88], SEM = 2.57°) and knee ROM was moderate (ICC_3,1_ = 0.61, 95% CI [0.23, 0.87], SEM = 2.50°).

There was a significant effect of wearing an exoskeleton during lifting on the ROM of the trunk, hip, and knee (F_1,4_ = 4.83, *p* = 0.031, ηp^2^ = 0.06). There was no significant interaction between wearing the exoskeleton and the ROM of the trunk, hip, and knee during lifting (F_2,8_ = 2.29, *p* = 0.12, ηp^2^ = 0.06). Wearing an exoskeleton significantly decreased knee ROM during lifting. However, wearing an exoskeleton did not significantly alter the ROM of the trunk and hip during lifting.

**Figure 4:**
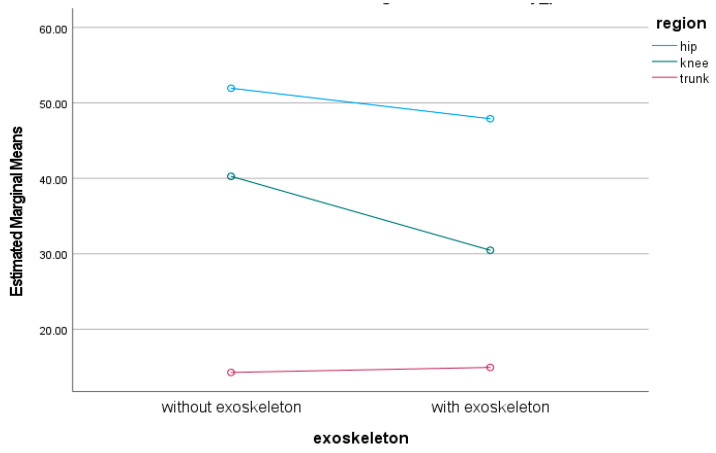
Trunk, hip and knee ROM with and without exoskeleton during lifting.

**Figure 5:**
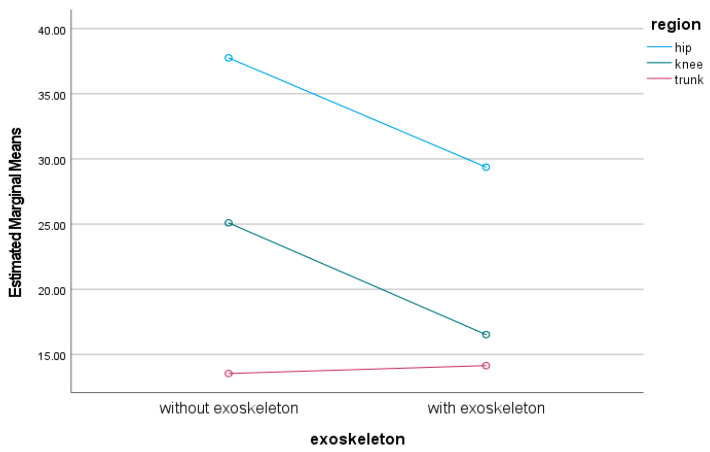
Trunk, hip and knee movement variation with and without exoskeleton.

The estimated marginal means of trunk, hip, and knee ROM and movement variability with and without the exoskeleton during lifting are shown in Figure 4 and Figure 5. There was a significant main effect of wearing an exoskeleton on trunk, hip, and knee movement variation during lifting (F_1,4_ = 11.13, *p* < 0.001, ηp^2^ = 0.13). There was a significant interaction between wearing exoskeleton and trunk, hip, and knee movement variation during lifting (F_2,8_ = 3.43, *p* = 0.037, ηp^2^ = 0.081). Post-hoc tests showed that hip (diff = −8.38, *p* = 0.045) and knee movement variation (diff = −8.57, *p* = 0.038) significantly decreased when lifting with an exoskeleton. However, trunk movement variability did not significantly change when lifting with an exoskeleton (diff = 0.60, *p* = 1.00).

## 4. Discussion

To the best of our knowledge, this is the first study that investigated how a novel minimally supportive lifting exoskeleton that provides support to the trunk, hip, and knee regions impacted lifting techniques (i.e., ROM of the trunk, hip, and knee, as well as movement variability) in people without LBP. This study found that our novel, minimally supportive lifting exoskeleton could produce reliable lifting movements for the trunk, hip, and knee seven days apart. Additionally, wearing this type of lifting exoskeleton, which supports the trunk, hip, and knee regions, resulted in a significant decrease in the ROM of the knee during lifting. Furthermore, wearing this lifting exoskeleton resulted in a significant decrease in hip and knee movement variations during lifting.

Our study’s findings suggest that wearing the novel exoskeleton during lifting produced reliable trunk, hip, and knee lifting movements across different days. These findings are consistent with past studies that utilized an exoskeleton to perform a lifting task in people without LBP [34]. However, it is worth noting that the weight that the participants lifted in Reneman et al. [34] were significantly heavier than our study (16 kg). Clinically, this reliable lifting movement could be desirable especially if it is associated with LBP symptom reduction in the short term and if this is associated with lower spinal load. Additionally, the current study found one participant had inconsistent trunk, hip, and knee movements each day.

Wearing an exoskeleton significantly decreased the ROM of the participants’ knees during lifting but did not significantly alter the ROM of the trunks and hips in people without LBP. This finding agrees with the previous literature [35]. Simon et al. [35] found that wearing an exoskeleton while lifting was associated with a 4.1% (2.6°) decrease in knee flexion ROM. In Simon et al.’s study [35], decreased ROM of the knee while wearing a lifting exoskeleton was found to be associated with increased biceps femoris muscle activity, decreased vastus lateralis muscle activity, and increased trunk extension torque, but no significant relationship with abdominal or back muscle activity. This could suggest that increased knee extension could be a desirable feature of an exoskeleton for people with LBP as it encourages hip extensor muscle activity to produce the trunk to extend and lift without significantly impacting the trunk’s muscle activity.

However, contrary to our findings, previous studies found an increase in knee movement of 22.9% (4.9°) while wearing an exoskeleton [36]. Luger et al. [36] proposed that boosted leg movement may occur as a result of force transmission by the passive joints from the trunk to the knees, implying that exoskeletons require a stronger leg posture modification to boost hip and knee movement. Contrary to these findings, most studies in the literature found that the exoskeleton did not alter knee movement. No change in knee movements as exoskeleton components were primarily designed to control the trunk movement [26,27]. Furthermore, Koopman et al. [27] argued that the exoskeleton components had limited performance, implying that exoskeletons did not support leg movement. Overall, the effects of the exoskeleton that support the trunk, hip, and knee may modify knee movements during lifting in people without LBP and should be investigated in future studies. Contrary to our expectations wearing an exoskeleton did not significantly alter trunk and hip ROM during lifting. Our findings were in line with previous literature that found that lifting the exoskeleton may not alter the movement of the trunk and hip [35]. No change in ROM can be a result of poor support from the passive components of the exoskeleton at these joints [37]. However, the previous literature found that exoskeletons can decrease movement of the trunk and knee [27,35]. They propose that when exoskeleton components are more tightly secured to the joints, they may prevent the joints from extending which leads to reduced flexion ROM. In contrast, there is evidence in the literature that exoskeletons can increase trunk and hip ROM during lifting [26,36]. This increase may result when tightly secured components of the passive exoskeleton have a greater range for flexion than the user’s maximum flexion ROM [38]. Therefore, a stronger exoskeleton trunk and hip component may contribute to changes in lifting movement.

In this study, wearing an exoskeleton that supports the trunk, hip, and knee joints during lifting was not associated with changes in trunk ROM. Wearing the exoskeleton was not associated with significant changes in trunk movement; this finding was in line with Simon et al. [35] who did not find changes in trunk movement when wearing an exoskeleton. The limited change in trunk movement is favorable to minimize restriction in back movement and force compensatory movement using the legs; however, contrary to this finding, Koopman et al. [27] reported a decrease in trunk ROM during lifting. Trunk ROM is limited due to contact by the exoskeleton components, particularly those that include trunk, chest, and hip components. There is evidence to suggest that exoskeleton components can overtake the user’s back movement (the user relaxes their trunk) and restrict their legs leading to reduced spinal loading (at L5-S1). Contrary to these findings, Abdoli-Eramaki et al. [26] found an increase in trunk ROM during lifting. This occurs when passive components of the exoskeleton activate after the user reaches maximum trunk flexion ROM [38]. Therefore, a stronger exoskeleton trunk component may contribute to back muscle movement (i.e., erector spinae). Exoskeleton components on the trunk may contribute to changes in trunk movement and should be considered in future testing with lifting exoskeletons.

The lifting exoskeleton used in this study, which supported the trunk, hip, and knee while lifting, resulted in decreased hip and knee movement variations in the participants who wore it. However, interestingly, wearing an exoskeleton did not significantly alter their trunks’ movement variability. These findings are in agreement with those reported in the literature [39]. Asgari et al. [39] found that wearing an exoskeleton while lifting was associated with a decrease in movement variability during lifting. Decreased movement variation may indicate greater stability from a lifting exoskeleton [40]. In comparison to commercially available lifting exoskeletons that used spring and elastic components, the novel exoskeleton resulted in a significant decrease in leg movement variability with only light minimal support components (i.e., no spring or elastic components). Studies have shown that wearing an exosuit exerts a force extending the leg joints causing reduced ROM and restrictive leg movement [35]. Therefore, a light minimal support exoskeleton is potentially sufficient to have an impact on people’s movement variability. There is emerging evidence that lower limb proprioception is decreased in people with LPB which could result in impaired balance [41]. Therefore, it is perhaps desirable to improve stability by decreasing movement variability in the lower limbs during lifting.

This is the first study that aimed to assess changes in lifting kinematics using a lifting exoskeleton that supports the trunk, hip, and knee simultaneously. Therefore, the current study provides the groundwork and primary findings for future studies to investigate minimal support lifting exoskeletons that support trunk, hip, and knee joints altogether. Findings from this study suggest that a minimal support exoskeleton can demonstrate reliable results one week apart and may limit lifting movement (i.e., ROM and movement variability) in people without LBP. The current study had several limitations that must be considered; it is still unclear if changes in muscle compressive forces of the spine and legs relative to lifting kinematics have an effect on trunk and leg health. Compressive forces should be tested as an increase or decrease in lifting ROM can be indicative of beneficial or aggravating effects on trunk and lower limb health [26]. Thus, future studies should investigate muscle, spinal, and compressive forces when wearing the new exoskeleton using EMG. Additionally, the effect of the minimally supportive exoskeleton is unclear. Powered exoskeletons have been shown to produce a greater decrease in trunk ROM than passive exoskeletons [42,43]. Furthermore, changes to ankle movement were not tested as the main con-tributing leg joints to lifting are the hip and knee [44]. However, recently exoskeletons have been shown to increase ankle dorsiflexion [35]. Moreover, the current pilot study recruited non-LBP participants and is unclear on its effect on the treatment of occupational LBP. Future studies should investigate the effect of minimal support exoskeleton on people with LBP and the potential to be a treatment for LBP [35,45].

The current literature suggests that wearing a lifting exoskeleton can result in an equivocal change in the trunk, hip, and knee ROM during lifting. Specifically, wearing an exoskeleton may increase or reduce trunk and lower limb ROM compared to not wearing an exoskeleton during lifting. Wearing a lifting exoskeleton resulted in increased trunk ROM in three studies [26,38,46] and decreased trunk ROM in five studies [27,42,43,45,47]. This may suggest that an exoskeleton with lumbar support may provide positive reinforcement or assurance to the wearer that could lead to increased trunk flexion-extension ROM. Facilitation of trunk ROM could be observed in lifting exoskeletons that placed their lumbar support around T7 to S1 vertebrae [26,38,46]. However, other studies discovered that exoskeletons with a trunk support can restrict lumber movement [27] due to the compression (i.e., bracing) of the thorax and abdomen caused by the close-fitting of rigid components in some exoskeletons [46]. Therefore, future lifting exoskeleton developers should consider the placement of lumbar support in the design of their exoskeletons as this will have a significant impact on trunk ROM. In addition, wearing a lifting exoskeleton could also result in either increased [36,45] or decreased hip and knee ROM [35]. Previous studies suggested that exoskeletons can encourage more movement in the hip and knee when the lumbar spine is blocked (i.e., tight fitting around the chest and abdomen) [45,48]. Therefore, due to the restrictions imposed on the trunk, this exoskeleton would require its wearer to adopt a squat lifting technique (i.e., lifting with the back upright and the lower limbs bent) to complete a lifting task [49]–hence, decreasing lifting movement variability. Although it was once thought that the squat lifting technique would decrease the amount of loading in the back, the recent literature has demonstrated that this is not true. In people with LBP, restricting trunk ROM could be a helpful short-term strategy to decrease symptom provocation via limiting lifting movements from reaching the extremes of trunk ROM. This, in turn, protects the joints and ligaments of the lumbar vertebra from increased irritation [50]. However, restoration of one’s available trunk ROM is an essential part of LBP rehabilitation [51]. Therefore, exoskeletons that limit trunk ROM could hinder optimum rehabilitation when used as a long-term solution. The design of future lifting exoskeletons should aim to restore appropriate lifting behavior that is context-dependent rather than pushing its wearer to adopt a particular lifting technique. Additionally, none of the studies included identified whether wearing an exoskeleton would yield a reliable lifting movement. This is crucial as the aims of wearing a lifting exoskeleton are ultimately to identify and guide its wearer to adopt an optimum lifting technique. Once identified, an optimum lifting technique must be reliably reproduced to minimize the risk of occupational injury. Additionally, within a rehabilitation framework, an exoskeleton could be utilized as a tool to bridge physical rehabilitation and return to work (i.e., a tool to facilitate gradual exposure to loading). Therefore, the use of some lifting exoskeletons, in many instances, is temporary. However, it is currently unknown whether wearing an exoskeleton would yield a short-term motor learning effect on its wearer. Having answers to these questions would help establish a lifting exoskeleton as a possible rehabilitation tool for people with lifting-related LBP.

## 5. Conclusions

Wearing the minimally supportive exoskeleton during lifting produced reliable trunk, hip, and knee lifting movements for participants across different days of this study. Wearing an exoskeleton significantly decreased their knees’ ROM during lifting but did not impact the trunk’s and hip’s ROMs. Movement variation with and without exoskeleton significantly decreased for the hip and knee but did not change for the trunk. These findings suggest that, while the exoskeleton can help standardize movement and potentially reduce variability, its effect on the knee’s ROM and movement variation may influence the risk of injury. Specifically, reduced knee ROM could potentially limit the movement needed for safe lifting, which may influence injury risk. However, the unchanged ROM of the trunk suggests that the exoskeleton does not fully address trunk stabilization, which is also critical in injury prevention. Future studies should aim to investigate the effect of wearing a minimally supportive exoskeleton on spinal loading in people with low back pain and investigate the viability of adding monitoring technologies, such as the IMU, to this lifting exoskeleton, which could monitor lifting movements at work or clinical settings.

## Figures and Tables

**Table 1:** Descriptive data of participant characteristics.

Variables (Unit)	People without LBP (*n* = 14) Mean ± SD
Age (years)	24 ± 4.51
Gender (%Male)	11 (79%)
Height (m)	1.70 ± 0.08
Mass (kg)	67.56 ± 8.32
BMI (kg/m^2^)	23.32 ± 2.62

LBP = Low Back Pain, BMI = Body Mass Index, SD = Standard Deviation.

**Table 2:** Descriptive data of participant kinematics.

Region	Without Exoskeleton (*n* = 14) Mean ± SD	With Exoskeleton (*n* = 14) Mean ± SD
Trunk (°)	14.28 ± 5.84	14.95 ± 6.57
Hip (°)	40.27 ± 10.04	30.47 ± 14.07
Knee (°)	51.95 ± 6.48	47.91 ± 9.23

SD = Standard Deviation.

## Data Availability

Data will be made available upon direct request to the corresponding author.
